# An exploratory study of adolescent response to fluoxetine using psychological and biological predictors

**DOI:** 10.7717/peerj.4240

**Published:** 2018-01-10

**Authors:** Ada H. Zohar, Tamar Eilat, Maya Amitai, Michal Taler, Romi Bari, Alon Chen, Alan Apter, Avraham Weizman, Silvana Fennig

**Affiliations:** 1 Graduate Program in Clinical Psychology, Ruppin Academic Center, Emek Hefer, Israel; 2 Graduate Psychology in Clinical Psychology, Ruppin Academic Center, Emek Hefer, Israel; 3 The Ruhman Family Laboratory for Research on the Neurobiology of Stress, Department of Neurobiology, Weizmann Institute of Science, Rehovot, Israel; 4 Psychological Medicine, Schneider Children’s Medical Center of Israel, Petah Tikva, Israel; 5 Sackler School of Medicine, Tel Aviv University, Tel Aviv, Israel; 6 Felsenstein Medical Research Center, Petah Tikva, Israel; 7 Department of Stress Neurobiology and Neurogenetics, Max-Planck Institute of Psychiatry, Munich, Germany; 8 Research Center, Geha Mental Health Center, Petah Tikva, Israel

**Keywords:** Fluoxetine, Adolescent, Treatment response, Ex post-facto study, Type D personality, Personality, Treatment response, SSRI

## Abstract

**Background:**

Not enough is known about predicting therapeutic response to serotonin-specific reuptake inhibitors, and specifically to fluoxetine. This exploratory study used psychological and biological markers for (retrospective) prediction of treatment-response to fluoxetine in depressed and/or anxious adolescents.

**Methods:**

Forty-one consecutive adolescent outpatients with a primary diagnosis of severe affective and/or anxiety disorders were assessed and treated with an open-label 8-week trial of fluoxetine. Type D personality was assessed with the 14-item questionnaire, the DS14. In addition, TNFα, IL-6, and IL-1b were measured pre- and post-treatment.

**Results:**

There was an elevation of Type D personality in patients, compared to the adolescent population rate. Post-treatment, 44% of patients were classified as non-responders; the relative risk of non-response for Type D personality patients was 2.8. Binary logistic regression predicting response vs. non-response showed a contribution of initial TNFα levels as well as Type D personality to non-response.

**Conclusions:**

In this exploratory study, the most significant contributor to non-response was Type D personality. However, the measurement of Type D was not prospective, and thus may be confounded with psychiatric morbidity. The measurement of personality in psychiatric settings may contribute to the understanding of treatment response and have clinical utility.

## Introduction

Although serotonin-specific reuptake inhibitors (SSRIs), and in particular fluoxetine, have revolutionized the treatment of depressive and anxiety disorders, there is a sizable minority of patients who do not improve during the course of treatment. It would be of great clinical utility to be able to predict which patients might profit from a course of fluoxetine and which patients might be better treated with a different medication or some other intervention. The problem is compounded by the 4–6-week delay in clinical response in those patients who do respond. The damage to those who turn out to be non-responders includes their persisting disorders and distress, their disappointment with the failure of the treatment they were given, and the decreased conditional likelihood of the efficacy of the next medication prescribed. In children and adolescents, there is an additional consideration the risk of self-injurious behavior activation related to SSRI treatment (Elbe et al., 2014).

Although there is some argument about the relative importance of the placebo response and the actual clinical effect of fluoxetine and other SSRIs, there is general agreement that fluoxetine is the best first choice medication for depression and anxiety. The position paper of child and adolescent psychiatry for the Canadian academy (Garland et al., 2016) concludes by saying that “the risk-benefit balance for fluoxetine in child and youth depression is favorable” (p 6). Walkup (2017) comes to a similar conclusion and moreover, he estimates, based only on publicly funded research, that fluoxetine adds 25–30% to the response rate for depressed children and adolescents, beyond the 30–35% placebo response rate.

By all standards, then, fluoxetine is the first drug of choice, but by the best estimates, a sizable minority of children and adolescents, about a third, will not respond to this medication.

Type D, or “distressed” personality, was posited by Denollet (2005). It has two component traits, social inhibition (SI) and negative affectivity (NA). Type D individuals are less likely to self-disclose and to share their medical symptoms with their doctors (Schiffer et al., 2007), probably due to their discomfort with and distrust of others (SI). They are more alexythemic than non-D individuals (Zohar et al., 2011; Batselé et al., 2016). However, they are awash with negative affectivity (NA), especially moodiness and irritability. Owing to their SI, Type D individuals are less likely to seek or to profit from social support even when feeling bad. Thus the elevation of both SI and NA is the source of this enduring pattern of distress.

Being Type D is associated with increased risk for worse mental health conditions (Lee et al., 2012), including depression and anxiety. Type D personality is associated with anxiety and depression in adult cardiac patients (Kupper et al., 2013) and in non-patients in the community (Zohar et al., 2011). Type D is also associated with elevated clinical depression in adolescent out-patients (Zhang, Li & Zou, 2011) and with an elevation of symptoms of depression and anxiety in adolescents in the community (Lee et al., 2012).

At least two pathways have been suggested between personality Type D and ill-health: overactivation of the immune system and poor health behavior.

D’Acquisto (2017) suggests that negative affect and immune activation are potential responses to perceived immunological threat and/or perceived emotional stress, and that they have a synergetic effect on each other. In a large scale empirical study, Van Dooren et al. (2016) measured plasma biomarkers of inflammation and endothelial dysfunction as well as Type D and depression. Van Dooren et al. (2016) found that cytokine levels, and markers of endothelial dysfunction were related to Type D personality, and to depression, and that Type D personality had a contribution to the prediction of depression over and beyond that of the biomarkers.

Another possible connection between Type D on the one hand and depression and anxiety disorders on the other is the relative self-neglect and failure to adopt health behaviors. Zohar et al. (2011) found that Type D individuals reported less sexual activity and less subjective health; Kupper et al. (2013) found that Type D individuals were more likely to smoke and to have sedentary lifestyles; Williams, Abbott & Kerr (2016) found that Type D individuals were less likely to engage in health behaviors including eating a balanced diet, sleeping well, and getting enough physical activity.

The current study set out to identify predictors of treatment response of adolescents with affective/and or anxiety disorders to fluoxetine. The role of personality was assessed using the Type D measure because of its known association with these disorders, and because of its relationship to hyperreactivity of the immune system.

## Methods

### Participants—clinical group

Forty-one consecutive outpatient treatment-naïve adolescents, mean age 14.12, 53.7% male, were referred to the fluoxetine trial in a tertiary medical center. They were diagnosed with affective and anxiety disorders, as well as with comorbid disorders. Most (59%) met criteria for major depression and the remainder met criteria for an anxiety disorder. Nearly all the patients (92.7%) had at least one comorbid diagnosis. The mean number of co-morbid disorders was 3.7 (SD 1.4). Exclusion criteria were intellectual disability, autistic spectrum disorder, mania or hypomania, psychosis, substance use, a neurological disorder, or a major medical disease. Inclusion criteria were a major depression or anxiety disorder with CGI-S ≥ 4, i.e., of at least moderate severity. A bi-weekly clinical assessment ensured tolerability.

### Participants—community volunteer comparison group

Adolescents attending a high school were approached to participate in the pilot study. Seventy adolescents between the age of 12 and 18, of whom 34 (48.7%) were boys, completed the DS14.

### Ethical considerations

The study protocol was approved by the ethics committee of Schneider’s Children Hospital, where the study took place (IRB approval #0097-11-RMC). Informed consent with supporting information was signed by parents of minors, and for adolescents over 18, by the participants themselves. No funding was received from funding agencies, in the public, commercial or non-for profit sectors for this study.

### Measures and procedure

#### Personality

Type D was assessed using the DS14 (Denollet, 2005) in Hebrew (Zohar et al., 2011). The DS14 has two 7-item subscales, social inhibition (SI) and negative affectivity (NA). A score of 10 or more on both subscales classifies an individual as Type D, or else individuals are classified as Non-D. As the DS14 was written for adults, slight language modification was introduced for five of the items so as to relate explicitly to age peers. This version of the DS14 was then piloted on 70 adolescents in the community, 12–18 years of age. It took about 5 min to complete and was well understood and tolerated. The prevalence of Type D in the community sample was 21.4% ± 5, no different from the prevalence for adults in the community 24.1% ± 1 (Zohar et al., 2011). DS14 was administered independently of the treatment team, and Type D status was only revealed after post-treatment evaluations were completed.

#### Clinical status evaluation

Subjects were assessed by the attending physician five times: at intake, and at two, four, six and eight weeks. Diagnostic evaluation was conducted using the Hebrew version of the Schedule for Affective Disorders and Schizophrenia for School-Age Children–Present and Lifetime version (K-SADS-PL) (Kaufman et al., 1997), thus diagnoses were given according to DSM-IV categories.

Overall clinical severity and improvement were assessed using the CGI-Severity and CGI-Improvement (CGI-I) subscales.

Continuous measures of depression and anxiety were obtained using the Children’s Depression Rating Scale–Revised (CDRS-R) (Poznanski et al., 1984), the Beck Depression Inventory (BDI) (Smucker et al., 1986), and the Screen for Child Anxiety Related Emotional Disorders (SCARED) (Birmaher, Brent & Benson, 1998; Birmaher et al., 1999). The scales were administered three times for each patient: at outset, midtrial (four weeks) and trial completion (eight weeks).

#### Cytokine measurement

Blood samples were collected pre and post treatment between 9 a.m. and 11 a.m. Tumor necrosis factor (TNF)-α, interleukin (IL)-6, and IL-1β were measured. A detailed description of the methodology for immunological markers is given in Amitai et al. (2016).

#### Medication

The dose was 20/g for responders and 40 mg/d for non-responders or partial responders. Compliance was based on the reports of the patients and their parents.

The parent-on-child reports and the child reports were collected by one research assistant (RB), the Type D assessment by another member of the research team (TE). All clinical assessments were conducted by the attending physician (MA). The assessors did not share information during the trial, and the data was integrated for analysis after the trial completion.

### Data analysis

The different assessments and test results were entered into one SPSS data file in preparation for the current study and checked for correctness. All Analyses were carried out in SPSS for Windows (version 23) and included correlations, *t*-test for group comparison, cross-tabulation with Chi-square, and logistic regression.

## Results

### Participant characteristics at intake

The participants were assessed at intake by the attending physician and the research assistants. Table 1 shows their status at the outset.

**Table 1:** Characteristics of study participants at baseline.

Variable	Count (%)/mean ± SD
Gender	22 boys (53.6%)
Age	14.1 ± 2.3
Major depressive disorder	30 (73.2%)
Panic disorder	11 (26.8%)
Generalized anxiety disorder	22 (53.6%)
Post-traumatic stress disorder	1 (2.4%)
Obsessive compulsive disorder	9 (21.9%)
Separation anxiety	12 (29.3%)
Social phobia	19 (46.3%)
Specific phobia	22 (53.6%)
Overall number of diagnoses	3.7 ± 1.4
BDI	18.3 ± 9.8
CDRS	57.4 + 20.2
SCARED-child	29.8 ± 12.4
SCARED-parent	28.7 ± 15.7
CGI	5.1 ± 0.5

**Notes.**

Categorical diagnoses are according to DSM-IV.

BDI Beck depression inventory, subscript denotes week of the study CDRS Child depression rating scale–revised SCARED-child Screen for Child Anxiety Related Emotional Disorders self-report SCARED-parent Screen for Child Anxiety Related Emotional Disorders parental report CGI Clinical Global Impression

### Type D personality in adolescent patients versus community controls

Of the 41 patients, 27 (66%) scored 10 or more on both DS14 subscales, meeting criteria for Type D personality, while of the 70 controls, 15 (21%) had Type D personality; this was a significant elevation, ${\chi }_{(1)}^{2}=12.3$, (*p* < 0.001). In comparison with an adult Israeli community sample (Zohar et al., 2011) in which 326 out of 1,350 (24.1%) met criteria for Type D the rate in this patient sample was also elevated, ${\chi }_{(1)}^{2}=19.9$, (*p* < 0.0001).

### Type D personality and symptom severity at baseline

The patients with and without Type D personality were compared for symptom severity pre-treatment. For the Clinician Global Severity scale, for the SCARED by child self-report and by parental report, there were no significant differences. However, for depressive symptoms, the Type D patients had significantly elevated scores: for the CDRS Type D patients had a mean of 67.52 (*SD* = 16.4) while the Non D patients had a mean of 50.16 (*SD* = 19.8; *t* =  − 2.95, *p* < 0.005). For the BDI Type D patients had a mean of 24.0 (*SD* = 9.3) while the Non D had a mean of 14.3 (*SD* = 8.2; *t* = −.3.49, *p* < 0.001). Because of the initial greater depression symptom severity of the Type D adolescents, baseline depression was controlled for subsequent analyses.

### Relationship between the DS14 scale scores and depressive symptoms

Further investigation of the relationship between the scale scores and the depression inventories was conducted by calculating the correlations between the scale scores and the measures of depression at baseline, midway through the treatment period and at treatment termination. The correlations are shown in Table 2 below. Both subscales correlate significantly and consistently with the BDI and CDRS-R scales scores, underscoring the relationship between Type D personality and depression; in particular the NA scale of the DS14.

**Table 2:** DS14 subscales NA and SI’s correlations with measures of depressive symptoms.

	BDI_0_	BDI_4_	BDI_8_	CDRS_0_	CDRS_4_	CDRS_8_
NA^**^	.595	.664	.615	.481	.636	.570
SI^*^	.334	.312	.366	.091	.333	.366

**Notes.**

BDI Beck depression inventory, subscript denotes week of the study CDRS Child depression rating scale–revised, subscript denotes week of the study

* SI and CDRS_0_ were not correlated significantly, all other correlations of SI with depression scales were significant at *p* < 0.01.

** All correlations with NA were significant at *p* < 0.0001.

### Type D personality and cytokine levels

Comparison of Type D and Non D patients for plasma level of TNFα, IL-6, and IL-1b, before and after treatment, showed no significant differences for any of the cytokines measured, before or after treatment, nor were the difference-scores for any of the three cytokines significantly different in the two patient groups. Thus, no association was shown between personality and hyperactivity of the immune system.

### Predicting response status from patient characteristics

The patients were assessed by the attending psychiatrist at the conclusion of the trial and coded as responders vs. non-responders. The responders were 14.7 ± 2.2 years of age, and the non-responders were 13.7 ± 2.3 years of age (*t* = 1.3, NS); the number of co-morbid diagnoses of the responders was 3.7 ± 1.4, and of the non-responders 3.8 ± 1.4 (*t* =  − 0.26, NS). There were eight boys (44%) among the non-responders and 14 (61%) among the responders, but this also did not constitute a significant difference (*χ*^2^ = 1.1, NS). The DS14 scores of the groups were very different; for responders the SI subscale score was 8.3 ± 4.3 and for non-responders it was 17.1 ± 6.3 (*t* =  − 5.2, *p* < 0.001); for responders the NA subscale score was 4.8 ± 6.4 and for non-responders it was 16.1 ± 4.9 (*t* =  − 6.1, *p* < 0.001). To test the effect of the other patient characteristics on the outcome, a binary logistic regression was performed, with the following predictors: first block, age and sex as control variables; second block, baseline depressive symptoms; third block, baseline TNFα levels and last Type D status. These results are shown in Table 3.

**Table 3:** Summary of logistic regression analysis for variables predicting response status to fluoxetine.

Block	Predictor	Beta	*t*	*p*	Fchange	Rchange
Baseline depression	BDI	−0.104	−0.57	0.585	0.93	0.046
	CDRS	0.328	2.06	0.047		
Cytokynes	TNFα	0.357	−2.63	0.013	6.82	0.147
Personality	Type D	0.541	−3.34	0.002	11.17	0.186

**Notes.**

Age and Gender were run as controls and are not shown in the table.

BDI Beck Depressive Inventory CDRS Children’s Depression Rating Scale

For the complete model, *F* = 4.339, *p* = 0.002, *R*^2^ = 0.434, *adjR*^2^ = 0.334.

As can be seen in Table 3, initial TNFα level contributed to non-response, and Type D personality status contributed to non-response over and beyond all the other predictors, including the initial depressive symptoms.

### Type D and non D patients’ clinical assessment post-treatment

The patients with and without Type D personality were compared for symptom severity post-treatment. For all measures Type D patients were significantly elevated versus non-D patients. The results are summarized in Table 4.

**Table 4:** Type D personality and post treatment clinical status.

	Type D (*N* = 17)		Non-D (*N* = 24)		*t*	*p*
	M	SD	M	SD		
SCARED child	29.23	16.9	16	12.1	−2.914	0.003
SCARED parent	26.58	18.3	18.16	10.8	−1.846	0.036
CDRS-R	43	15.6	29.2	10.2	−3.416	0.0005
BDI	15.94	10.1	5.95	8.6	−3.394	0.001
CGI	3.31	1	2.77	0.6	−1.96	0.029

**Notes.**

SCARED Screen for Child Anxiety Related Emotional Disorders CDRS-R Children’s Depression Rating Scale–Revised BDI Beck Depression Inventory CGI Clinical Global Impressions-Severity

### Type D personality and responder status

Of the 41 patients, 23 (56.1%) were classified by the attending physician (M.A.) as responders. Of the responders 5 (of 17) were Type D and 18 (of 24) were Non D. The Relative risk of non-response for Type D patients was thus 2.82 (95% CI [1.3–6.0]).

## Discussion

When considering the results of this study, its limitations should be kept in mind. In particular, this study includes a relatively small patient sample, and the fluoxetine trial was open label, not allowing for the assessment of placebo response. In addition, although Type D personality contributed to the prediction of non-response, its association with depressive symptoms was very significant, and the question of its temporal stability and the best time to measure personality status vs. clinical status has not been studied in a satisfactory way in the context of adolescent depression. Further studies, with a prospective design, and using large patient samples, are required to confirm our results.

The current study found a significant elevation of Type D, or distressed personality, in a clinical sample of depressed and/or anxious adolescents of psychiatric outpatients relative to community controls. This finding is consistent with previous research. In their systematic review, Mols & Denollet (2010) concluded that there was evidence for the association of Type D personality with both depression and anxiety disorders. Zhang, Li & Zou (2011) reported an elevation of Type D personality in depressed Chinese adolescents relative to healthy controls. In a prospective study, Doyle et al. (2011) found that the risk of developing depression within a year of hospitalization for acute coronary syndrome was more than three-fold for Type D personality patients. Van Dooren et al. (2016) found in an adult community sample, that Type D was associated with a 13-fold increased risk of meeting criteria for depression. However, there is a clear relationship between scoring high on both subscales of the DS14, required for classification as Type D, and depressive symptoms. This relationship raises doubt as to the stability and independence of the personality status vs. the clinical assessment. If Type D personality is associated with depression it would not be a predictor of the disorder but an indicator of its presence. Ossola et al. (2015) found, in a study of patients presenting with acute cardiac syndrome, that the NA subscale of the DS14 was so strongly associated with depressive symptoms that Type D was better conceived of as a state description than as a stable personality typology. The current study also found strong correlations between both depressive subscales used and the NA subscale of the DS14 at all three time-points.

Another related issue in using Type D status as a predictor is that of its temporal stability. The typology (i.e., classifying individuals into Type D and Non-D) is defined by scoring 10 or more on both of the DS14 subscales. Thus gaining or losing one point on either of the subscales due to an individual’s actual change or due to measurement error is enough to change the personality status. In a community sample of older adults who self-reported on the DS14 at baseline and six years later, 56.9% of individuals who were Type D at time2 had been Type D at time1 (Zohar, 2016). For children in the community (Jellesma, 2008) measured twice at a 18-month interval only 42.9% of those who were Type D at time2 had been Type D at time1. The temporal stability of caseness is considerably lower in cardiac patients. Ossola et al. (2015) found that less than a third of cardiac patients retained their Type D classification 12 months after an acute cardiac event. In using Type D as a predictor of health events, we lack a theoretical or empirical criterion for when Type D should be measured to ensure that it predates the outcome and is not just a description of the outcome. It should be noted that in the current study the differences in the DS14 scores between responder and non-responders were so large that it seems unlikely that temporal stability over the eight week trial constitutes an alternate explanation; however, to discriminate trait and state it would have been much more convincing if personality had been measured prospectively before the patients started exhibiting depression and anxiety.

Keeping these reservations in mind, the current study found that clinically depressed or anxious adolescents who have Type D personality had a nearly three-fold greater risk of non-response to the fluoxetine trial in this study. Increased risk remained for Type D personality patients to be non-responders even when controlling for initial depressive symptoms and for initial TNFα levels as shown in the binary logistic regression.

Overall, Type D patients in the current study presented more severe depression at baseline. At treatment termination, Type D adolescents were significantly worse off on all the clinical measures, anxiety scales, depression scales, and clinician rating scale. Thus there was a strong association between personality type, and fluoxetine trial outcome. This result is consistent with other medical procedures in which Type D individuals show less favorable outcomes. Zhang et al. (2016) found that Type D rectal cancer patients were less likely to respond positively to oncological treatment.

What is it about Type D personality that might be associated with less treatment response? It may be that the chronic distress of negative affectivity and social inhibition typical of Type D individuals puts them at risk of less effective immune response, and chronic inflammatory processes. This theory has extensive empirical support (Van Dooren et al., 2016; Jandackova et al., 2017). The current study measured three bio-markers of inflammatory processes, TNFα, IL-6, and IL-1b, which were measured pre- and post-treatment. Although there was a negative association between initial TNFα levels and treatment response (Amitai et al., 2016) there no association between TNFα levels and Type D personality. It does not seem that hyperactive immune function will explain the full force of association between Type D personality and non-response to fluoxetine in the current study.

An additional hypothesis proposed for the association of Type D personality with many bad health outcomes, is that Type D individuals are less likely to engage in health behavior vs. Non-D individuals. Williams et al. (2008) showed that Type D individuals were less likely to eat reasonably, to spend time outdoors every day, or to get a regular medical checkup. Williams, Abbott & Kerr (2016) showed that young adults who are Type D are less likely to engage in a healthy lifestyle and that these behaviors partially mediate the effect of Type D on self-reported (ill) health. There is also research supporting the notion that Type D individuals with cardiac conditions are less likely to complain of relevant symptoms to their physicians (Schiffer et al., 2007) and less likely to adhere to their cardiac medication (Williams et al., 2011). There is no such research on adolescent patients, and yet there is no reason to believe that adolescents with Type D personality would have as much health behavior as Non-D adolescents. It is possible that the Type D adolescents enrolled in the current study were less open with the treatment team about their relevant symptoms, or that they were less adherent to the treatment regime, or that they engaged in less health promoting behaviors that might potentially synergize the effect of the medication. More research is needed in order to understand the mechanisms at play.

## Conclusions

The additional risk conferred on adolescent patients who have Type D personality suggests that taking personality assessment into consideration while treating with psychotropic medication in a medical setting might improve treatment and overall therapeutic outcome. Personality assessment in medical settings is not usually included for practical reasons. The current study used a very brief and easily scored personality assessment, thus not adding to participant burden. A bigger potential barrier for including personality assessment in medication trials has to do with disciplinary lines between medicine and psychology. The strong results associated with the personality assessment in the current study suggest that this mixed strategy might be of clinical utility and might help to expand our understanding of the processes of drug response.

##  Supplemental Information

10.7717/peerj.4240/supp-1 Data S1 SPSS data file

## Additional Information and Declarations

### Data Availability

The following information was supplied regarding data availability: The raw data has been provided as a Supplemental File.
